# Depression and anxiety among older people in central africa: Epidemca population-based study

**DOI:** 10.1192/j.eurpsy.2021.639

**Published:** 2021-08-13

**Authors:** A. Gbessemehlan, M. Guerchet, C. Adou, J.-P. Clément, B. Ndamba-Bandzouzi, P. Mbelesso, D. Houinato, P.-M. Preux

**Affiliations:** 1 Inserm, Chu Limoges, Ird, U1094 Tropical Neuroepidemiology, Institute Of Epidemiology And Tropical Neurology, Geist, University of Limoges, Limoges, France; 2 Department Of Neurology, Brazzaville University Hospital, Brazzaville, Congo, Republic of; 3 Department Of Neurology, Amitié Hospital, Bangui, Central African Republic

**Keywords:** Depression, African Older people, Anxiety, EPIDEMCA

## Abstract

**Introduction:**

The burden of depression and anxiety is poorly documented in Central African populations.

**Objectives:**

To present the epidemiology of depressive and anxiety disorders among older people in two Central African countries.

**Methods:**

A cross-sectional population-based study was carried out in Republic of Congo (ROC) and Central African Republic (CAR) between 2011 - 2012 among people aged ≥ 65 years (EPIDEMCA study). Data were collected using a standardized questionnaire and participants underwent a brief physical examination. Depression and anxiety symptoms were ascertained using a community version of the Geriatric Mental State (GMS-B3). Probable cases were defined as having a GMS-AGECAT score ≥ 3. Logistic regression models were used to investigate the association between potential risk factors collected and presence of at least one of both symptoms.

**Results:**

Overall 2002 participants were included in the EPIDEMCA study. Median age of the participants was 72 years [interquartile range: 68 – 78 years] and 61.8% were females. Prevalence was 38.1% (95% Confidence Interval: 35.9% - 40.2%) for depression, 7.7% (95% CI: 6.5% - 8.9%) for anxiety. In total 40.1% had least one of both symptoms. In multivariable models, the following factors were associated with the presence of at least one of both symptoms: female sex, residence area, frailty, cognitive disorders, a high happiness score (protective) and hypertension (adjusted Odds Ratios from 1.3 to 1.7; p<0.01).

**Conclusions:**

In light of the high prevalence of both psychiatric symptoms among Central African older people, evidence on their epidemiology is important for better management and policy planning.

